# Inference for One-Way ANOVA with Equicorrelation Error Structure

**DOI:** 10.1155/2014/341617

**Published:** 2014-06-19

**Authors:** Weiyan Mu, Xiaojing Wang

**Affiliations:** School of Sciences, Beijing University of Civil Engineering and Architecture, Beijing 100081, China

## Abstract

We consider inferences in a one-way ANOVA model with equicorrelation error structures. Hypotheses of the equality of the means are discussed. A generalized *F*-test has been proposed by in the literature to compare the means of all populations. However, they did not discuss the performance of that test. We propose two methods, a generalized pivotal quantities-based method and a parametric bootstrap method, to test the hypotheses of equality of the means. We compare the empirical performance of the proposed tests with the generalized *F*-test. It can be seen from the simulation results that the generalized *F*-test does not perform well in terms of Type I error rate, and the proposed tests perform much better. We also provide corresponding simultaneous confidence intervals for all pair-wise differences of the means, whose coverage probabilities are close to the confidence level.

## 1. Introduction

The generalized *p* value approach and the generalized pivotal quantities introduced by Tsui and Weerahandi [1] and Weerahandi [2], respectively, have proven to be useful tools to study repeated measures models when the error variances are unequal. Inferences for many important linear models with unequal error variances have been discussed by these approaches (see Weerahandi [3] and references therein). For the problem of comparing the means of several populations with unequal variances, Weerahandi [4] introduced a generalized *F*-test using the notion of generalized *p* values. Since then the generalized *F*-test has been widely used to test the equality of the means or the fixed effects of ANOVA models (Ananda and Weerahandi [5], Gamage and Weerahandi [6], Bao and Ananda [7], and Mu et al. [8]) and other repeated measures models under heteroscedasticity (Chi and Weerahandi [9], Weerahandi and Berger [10], Lin and Lee [11], Ho and Weerahandi [12], and Mu and Xu [13]).

The ANOVA models are basic but important among linear models. Extensive studies have been done on the ANOVA models with usual independent error structure, even under heteroscedasticity (see, for example, Krishnamoorthy et al. [14] and references therein). However, it is often seen that the error variance varies over time in practical work. Therefore, it is required to assume more complicated error structures for investigating such situations. Lin and Lee [11] considered an ANOVA model with equicorrelation error structure under heteroscedasticity which is described as follows. Suppose a random sample of size *n*_*i*_ is available from the *i*th population, *i* = 1,…, *I*. Let *Y*_*ij*_, *i* = 1,…, *I*, *j* = 1,…, *n*_*i*_, be the random vectors of order *T* representing the observations taken from the *I* populations. We assume that the random vectors are all mutually independent and that we have a complete data set from all subjects in each group. Then, the model can be formulated as

(1) $$\begin{array}{c} Y_{ij}=\mu_{i}1_{T}+\alpha_{i}1_{T}+\varepsilon_{ij},\;i=1,\ldots ,I,\;\;j=1,\ldots ,n_{i}, \end{array}$$

where the error term *ε*_*ij*_ ~ *N*(0, Σ_*ei*_) with Σ_*ei*_ = *σ*_*i*_^2^[(1 − *ρ*)*I*_*T*_ + *ρ*1_*T*_1_*T*_′], and the random effects *α*_*i*_ ~ *N*(0, *σ*_*α*_^2^).

Lin and Lee [11] provided the generalized *F*-test to test the equality of the means of the *I* populations. However, this generalized *F*-test was not evaluated by simulation in their article. Note that the generalized *F*-test for the model with independent error structure has poor size performance when the group number is large (Krishnamoorthy et al. [14]). Based on the generalized pivotal quantities derived from a general method provided by Hannig et al. [15], we provide a new standardization-based *p* value for testing the equality of the means. A parametric bootstrap test following Krishnamoorthy et al. [14] is also presented. Furthermore, simultaneous confidence intervals of all pair-wise difference of the means are often important in practical work. We also present such simultaneous confidence intervals. Simulation results are reported in this paper and show that the Type I errors of the generalized *F*-test exceed the nominal level in many cases (similar to the case of independent error structure shown in Krishnamoorthy et a.l [14]), while the proposed tests and simultaneous confidence intervals have good frequentist properties under various sample size and parameter combinations.

The rest of the paper is organized as follows.  Section 2 discusses testing the equality of the means of the *I* populations. The generalized *F*-test, the standardization-based *p* value test, and the parametric bootstrap test for the hypotheses of the equality of the means are described in this section.  Section 3 provides the simultaneous confidence intervals for all pair-wise differences of the means. Simulation results for the Type I error probabilities of these three tests and the coverage probabilities of the simultaneous confidence intervals are provided in Section 4.  Section 5 shows an illustrative example and some concluding remarks are presented in Section 6.

## 2. Tests of the Means in One-Way ANOVA

In this section, we consider the problem of testing the equality of means:

(2) $$\begin{array}{c} H_{0}:\;\mu_{1}=\mu_{2}=\cdots =\mu_{I}⟷H_{1}:\;\text{not}\;\;\text{all}\;\;\mu_{i}\;\;\text{are}\;\;\text{equal}. \end{array}$$

Lin and Lee [11] gave the generalized *F*-test to deal with this problem. In the following, we describe the generalized *F*-test and two new tests.

### 2.1. The Generalized *F*-Test

We now describe the generalized *F*-test. From (1), Cov(*Y*_*ij*_) = Σ_*i*_ with

(3) $$\begin{array}{c} \Sigma_{i}^{-1}={\left[\sigma_{i}^{2}\left(1-\rho \right)\right]}^{-1}\left[I_{T}-\frac{\phi_{i}^{2}-\sigma_{i}^{2}\left(1-\rho \right)}{T\phi_{i}^{2}}1_{T}1_{T}^{\prime }\right], \end{array}$$

and *ϕ*_*i*_^2^ = *σ*_*i*_^2^(1 − *ρ*) + *T*(*ρσ*_*i*_^2^ + *σ*_*α*_^2^). Consequently, it can be obtained that the residual sum of squares

(4) $$\begin{array}{c} S_{i}^{2}=T\overset{n_{i}}{\underset{j=1}{\sum }}{\left({\bar{Y}}_{ij\cdot }-{\bar{Y}}_{i\cdot \cdot }\right)}^{2},\;i=1,\ldots ,I, \end{array}$$

is distributed as

(5) $$\begin{array}{c} V_{i}=\frac{S_{i}^{2}}{\phi_{i}^{2}}\sim \chi_{n_{i}-1}^{2}, \end{array}$$

where ${\bar{Y}}_{ij\cdot }=\sum_{t=1}^{T}1_{T}^{\prime }Y_{ijt}/(Tn_{i})$, and ${\bar{Y}}_{i\cdot \cdot }=\sum_{j=1}^{n_{i}}{\bar{Y}}_{ij\cdot }/n_{i}$. Write *N* = ∑_*i*=1_^*I*^ *n*_*i*_ and then

(6) $$\begin{array}{c} V=\overset{I}{\underset{i=1}{\sum }}V_{i}\sim \chi_{N-I}^{2}. \end{array}$$

Denote the standardized between-group sum of squares by

(7) $$\begin{array}{c} {\tilde{S}}_{B}^{2}={\tilde{S}}_{B}^{2}\left(\phi_{1}^{2},\ldots ,\phi_{I}^{2}\right)=\overset{I}{\underset{i=1}{\sum }}\frac{Tn_{i}}{\phi_{i}^{2}}{\bar{Y}}_{i\cdot \cdot }^{2}-\frac{{\left(\sum_{i=1}^{I}\left(Tn_{i}/\phi_{i}^{2}\right){\bar{Y}}_{i\cdot \cdot }\right)}^{2}}{\sum_{i=1}^{I}\left(Tn_{i}/\phi_{i}^{2}\right)}. \end{array}$$

The observed value of ${\tilde{S}}_{B}^{2}$ is denoted by ${\tilde{s}}_{B}^{2}$.

The potential extreme region for *H*_0_ : *μ*_1_ = ⋯ = *μ*_*I*_ is

(8) $$\begin{array}{c} \left\{{\tilde{S}}_{B}^{2}\left(\phi_{1}^{2},\ldots ,\phi_{I}^{2}\right)⩾{\tilde{s}}_{B}^{2}\left(\frac{s_{1}^{2}}{S_{1}^{2}/\phi_{1}^{2}},\ldots ,\frac{s_{I}^{2}}{S_{I}^{2}/\phi_{I}^{2}}\right)\right\}. \end{array}$$

The observed sample point (*s*_1_^2^,…, *s*_*I*_^2^) of (*S*_1_^2^,…, *S*_*I*_^2^) falls on the boundary of this set. The generalized *p* value can be expressed as

(9) p=P{S~B2(ϕ12,…,ϕI2)⩾s~B2(s12S12/ϕ12,…,sI2SI2/ϕI2)}=P{S~B2(ϕ12,…,ϕI2)V⩾s~B2(s12V1/V,…,sI2VI/V)}=1−EB1,…,BI−1{FI−1,N−I        ×[N−II−1         ×{s~B2(λ12B1B2⋯BI−1,…,             λk2(1−Bk−1)Bk⋯BI−1             ,…,λI21−BI−1)}]},

where *F*_*I*−1,*N*−*I*_ is the cdf of the *F* distribution with degrees of freedom *I* − 1 and *N* − *I*. The expectation is taken with respect to the independent beta random variables:

(10) $$\begin{array}{c} B_{t}=\frac{\sum_{i=1}^{t}U_{i}}{\sum_{i=1}^{t+1}U_{i}}\sim \text{Beta}\left(\frac{\sum_{i=1}^{t}n_{i}-1}{2},\frac{n_{t+1}-1}{2}\right),\;t=1,\ldots ,I. \end{array}$$

### 2.2. The Standardization-Based *p* Value Test

In this subsection, we provide new *p* values based on Mahalanobis norm to test the hypotheses (2). Denote *μ* = (*μ*_1_,…,*μ*_*I*_)′. Note that the hypotheses (2) can be rewritten in the matrix form

(11) $$\begin{array}{c} H_{0}:H\mu =0,\;\;H_{1}:H\mu \neq 0, \end{array}$$

where

(12) $$\begin{array}{c} H=\left(\begin{array}{cccccc} 1 & 0 & 0 & \cdots & 0 & -1\\ 0 & 1 & 0 & \cdots & 0 & -1\\ \vdots & \vdots & \vdots & \ddots & \vdots & \vdots \\ 0 & 0 & 0 & \cdots & 1 & -1 \end{array}\right), \end{array}$$

is an (*I* − 1) × *I* matrix.

Denote ${\hat{\mu }}_{i}={\bar{Y}}_{i\cdot \cdot }$. We have

(13) $$\begin{array}{c} {\hat{\mu }}_{i}=\frac{1}{n_{i}}\overset{n_{i}}{\underset{j=1}{\sum }}{\bar{Y}}_{ij\cdot }=\frac{1}{Tn_{i}}\overset{n_{i}}{\underset{j=1}{\sum }}1_{T}^{\prime }Y_{ij}\sim N\left(\mu_{i},\frac{\phi_{i}^{2}}{Tn_{i}}\right). \end{array}$$

Thus, it is easy to obtain that

(14) Tniϕi2(μ^i−μi)~N(0,1)Si2ϕi2~χni−12,i=1,…,I.

For *i* = 1,…, *I*, define

(15) Zi=Tniϕi2(μ^i∗−μi)~N(0,1),Vi=Si∗2ϕi2~χni−12,

where ${\hat{\mu }}_{i}^{*}={\hat{\mu }}_{i}(Y^{*}),S_{i}^{*2}=S_{i}^{2}(Y^{*})$, and Y* = (*Y*_11_*,…, *Y*_1*n*_1__*,…, *Y*_*I*1_*,…, *Y*_*In*_I__*) is an independent copy of *Y* = (*Y*_11_,…, *Y*_1*n*_1__,…, *Y*_*I*1_,…, *Y*_*In*_*I*__). By the structural method provided by Hannig et al. [15] (see Theorem 2 of their article), we can get the following fiducial generalized pivotal quantities of *μ*_*i*_ and *ϕ*_*i*_^2^:

(16) Rϕi2=Si2ViRμi=μ^i−ZiRϕi2Tnii=1,…,I.

Denote the Mahalanobis norm of a vector *x* with respect to a positive definite matrix *A* by ||*x*||_*A*_; that is, ${||x||}_{A}=\sqrt{x^{\prime }A^{-1}x}$. Thus, the fiducial generalized pivotal quantity of *Hμ*, the parameter of interest, is *R*_*Hμ*_ = *HR*_*μ*_, where *R*_*μ*_ = (*R*_*μ*_1__,…,*R*_*μ*_*I*__)′. Thus, a reasonable ellipsoidal confidence region of *Hμ* can be given by

(17) $$\begin{array}{c} \left\{H\mu :{||H\mu -{\text{E}}^{*}\left(R_{H\mu }\right)||}_{\text{Co}{\text{v}}^{*}(R_{H\mu })}⩽v_{\alpha }\right\}, \end{array}$$

where E* and Cov* represent the expectation and covariance with respect to Y*, respectively, and *v*_*α*_ is the upper *α*th quantile of ||*Hμ*−E*(*R*_*Hμ*_)||_Cov*(*R*_*Hμ*_)_ given the observation *Y*. By the relationship between confidence regions and hypotheses testing, the corresponding *p* value for the hypotheses (11) is
(18)
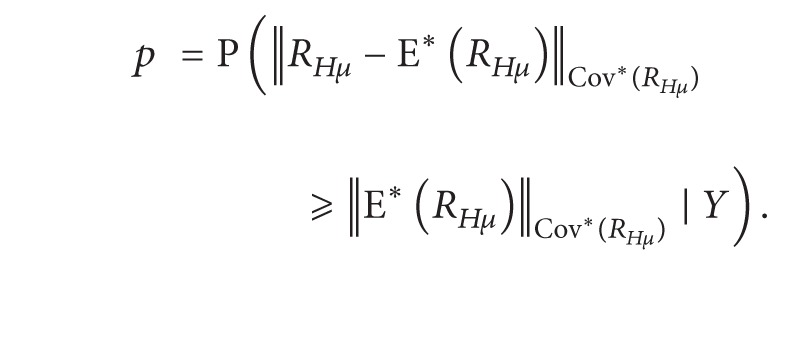

It is easy to show that ${\text{E}}^{*}\left(R_{H\mu }\right)=H\hat{\mu }$ and

(19) $$\begin{array}{c} \text{Co}{\text{v}}^{*}\left(R_{H\mu }\right)=H\text{Diag}\left(\frac{S_{i}^{2}}{Tn_{i}\left(n_{i}-3\right)}\right)H^{\prime }. \end{array}$$

This method for deriving generalized *p* values concerning vector parameters has been adopted in Xiong et al. [16].

### 2.3. The Parametric Bootstrap Test

Following Krishnamoorthy et al. [14], we present the parametric bootstrap test for (2). Denote ${\hat{\phi }}_{i}^{2}=S_{i}^{2}/(n_{i}-1)$. The test statistic is

(20) $$\begin{array}{c} \text{TS}={\tilde{S}}_{B}^{2}\left({\hat{\phi }}_{1}^{2},\ldots ,{\hat{\phi }}_{I}^{2}\right)=\overset{I}{\underset{i=1}{\sum }}\frac{Tn_{i}}{{\hat{\phi }}_{i}^{2}}{\hat{\mu }}_{i}^{2}-\frac{{\left(\sum_{i=1}^{I}\left(Tn_{i}/{\hat{\phi }}_{i}^{2}\right){\hat{\mu }}_{i}\right)}^{2}}{\sum_{i=1}^{I}\left(Tn_{i}/{\hat{\phi }}_{i}^{2}\right)}. \end{array}$$

We use the bootstrap method (Efron [17]) to approximate its null distribution. Generate the bootstrap sample ${\hat{\mu }}_{i}^{B}\sim N(0,{\hat{\phi }}_{i}^{2}/(Tn_{i}))$ and $(n_{i}-1){\left({\hat{\phi }}_{I}^{B}\right)}^{2}/{\hat{\phi }}_{i}^{2}\sim \chi_{n_{i}-1}^{2}$, for *i* = 1,…, *I*. The bootstrap version of TS is

(21) $$\begin{array}{c} {\text{TS}}^{B}=\overset{I}{\underset{i=1}{\sum }}\frac{Tn_{i}}{{\left({\hat{\phi }}_{i}^{B}\right)}^{2}}{\left({\hat{\mu }}_{i}^{B}\right)}^{2}-\frac{{\left(\sum_{i=1}^{I}\left(Tn_{i}/{\left({\hat{\phi }}_{i}^{B}\right)}^{2}\right){\hat{\mu }}_{i}^{B}\right)}^{2}}{\sum_{i=1}^{I}\left(Tn_{i}/{\left({\hat{\phi }}_{i}^{B}\right)}^{2}\right)}. \end{array}$$

Given the level *α*, we reject *H*_0_ if TS is greater than the conditional upper *α*th quantile of TS^*B*^ conditional on the observations. The quantile can be computed by the Monte Carlo method.

## 3. Simultaneous Confidence Intervals for the Means

For the case of independent error structure, Xiong and Mu [18] discussed simultaneous confidence intervals for all-pairwise differences of the means based on generalized pivotal quantities. Following their method, we now present such intervals under the model (1). Bootstrap simultaneous confidence intervals are also provided.

Denote

(22) $$\begin{array}{c} R_{d}=\underset{i<j}{\max}\left|\frac{R_{\mu_{i}}-R_{\mu_{j}}-{\text{E}}^{*}\left(R_{\mu_{i}}-R_{\mu_{j}}\right)}{{\left(\text{Va}{\text{r}}^{*}\left(R_{\mu_{i}}-R_{\mu_{j}}\right)\right)}^{1/2}}\right|, \end{array}$$

where

(23) $$\begin{array}{c} {\text{E}}^{*}\left(R_{\mu_{i}}-R_{\mu_{j}}\right)={\hat{\mu }}_{i}-{\hat{\mu }}_{j},\\ \text{Va}{\text{r}}^{*}\left(R_{\mu_{i}}-R_{\mu_{j}}\right)=\frac{S_{i}^{2}}{Tn_{i}\left(n_{i}-3\right)}+\frac{S_{j}^{2}}{Tn_{j}\left(n_{j}-3\right)}. \end{array}$$

Let *q*_*α*_ be the conditional upper *α*th quantile of *R*_*d*_. The distribution of the studentized maximum modulus statistic

(24) $$\begin{array}{c} M=\underset{i<j}{\max}\left|\frac{\left(\mu_{i}-\mu_{j}\right)-E^{*}\left(R_{\mu_{i}}-R_{\mu_{j}}\right)}{{\left(\text{Va}{\text{r}}^{*}\left(R_{\mu_{i}}-R_{\mu_{j}}\right)\right)}^{1/2}}\right|, \end{array}$$

can be approximated by the conditional distribution of *R*_*d*_ conditional on the observations. Then we have the following (1 − *α*) two-sided simultaneous confidence intervals for all-pairwise differences:

(25) μi−μj∈E∗(Rμi−Rμj)±qα(Var∗(Rμi−Rμj))1/2,∀i<j, i,j=1,…,I.

The bootstrap method approximates the distribution of *M* by the conditional distribution of *M* ^*B*^, the bootstrap version of *M* obtained by replacing ${\hat{\mu }}_{i}$ and ${\hat{\phi }}_{i}^{2}=S_{i}^{2}/(n_{i}-1)$ in *M* with ${\hat{\mu }}_{i}^{B}$ and ${\left({\hat{\phi }}_{i}^{B}\right)}^{2}$, where ${\hat{\mu }}_{i}^{B}\sim N({\hat{\mu }}_{i},{\hat{\phi }}_{i}^{2}/(Tn_{i}))$ and $(n_{i}-1){\left({\hat{\phi }}_{I}^{B}\right)}^{2}/{\hat{\phi }}_{i}^{2}\sim \chi_{n_{i}-1}^{2}$, for *i* = 1,…, *I*. Then, the bootstrap (1 − *α*) two-sided simultaneous confidence intervals are

(26) μi−μj∈μ^i−μ^j±qαB(Var∗(Rμi−Rμj))1/2,∀i<j, i,j=1,…,I,

where *q*_*α*_^*B*^ is the conditional upper *α*th quantile of *M* ^*B*^.

## 4. Simulation Studies

This section is devoted to evaluating the performances of the procedures described in this paper. We first consider comparing the generalized *F*-tests (denoted by GF), the standardization-based *p* value test (denoted by SP), and the parametric bootstrap test (denoted by PB). We will present the Type I error rates of the three tests through the Monte Carlo simulation. For a given sample size and parameter configuration shown in Table 1, the repetition is 5000 times. The Monte Carlo sample size for computing the *p* values is 5000. The nominal level is given as 0.05. We take the dimension *T* of *Y*_*ij*_ as 4 in our simulation.

The simulated Type I error rates of the two tests are reported in Table 1. The results are described as follows.

- From the results of the two cases, *ρ* = 0 and *ρ* = 0.5, when *I* = 6, we can see that *ρ* does not have significant influence on the Type I errors of the two tests.
- The Type I error rates of the GF test exceed the nominal level in most cases, especially for relatively large *I*. In the worst case of *I* = 10, the Type I error rate of the GF test is as large as 0.102.
- The SP test appears to be some conservative when the sample sizes are relatively small. As the sample sizes increase, its Type I error rates become quite close to the nominal level.
- The Type I error rates of the PB test sometimes exceed the nominal level but they are the closest among the three tests in most cases.

We have an overall conclusion that the proposed SP and PB tests perform significantly better than the GF test.

The simulated coverage probabilities of the proposed simultaneous confidence intervals (25) and (26) are shown in Table 2. It can be seen that the simulated coverage probabilities of such intervals are larger than and/or close to the confidence level under all sample size and parameter combinations.

## 5. An Illustrative Example

We will illustrate our methods using an example. The data in this example are generated assuming model (1) with *I* = 8, *T* = 4, *σ*_*α*_ = 1, and *ρ* = 0.2. We will compare the performances of the generalized *F*-test and the proposed tests with respect to Type I error in the one-way ANOVA model with unequal error variances. The problem of comparing seven means is considered. The mean of each distribution is taken to be 1 so that the null hypotheses *H*_01_ : *μ*_2_ = *μ*_3_ = ⋯ = *μ*_7_,…, *H*_08_ : *μ*_1_ = *μ*_2_ = ⋯ = *μ*_7_ are all true.  Table 3 shows the results of a simulated experiment in which data are generated from normal distributions with means 1 and the values of *n*_*i*_, *σ*_*i*_, *ϕ*_*i*_, and *i* = 1,…, 8. The treatment means ${\hat{\mu }}_{i}$ and residual sum of squares *S*_*i*_^2^ corresponding to each value of *ϕ*_*i*_ are also provided.

Seven treatments out of eight in Table 4 are compared at a time. The *p* values of the generalized *F*-test, the standardization-based test, and the parametric bootstrap test are denoted by *p*_GF_, *p*_SP_, and *p*_PB_, respectively. We compute them using 500,000 Monte Carlo samples. The results are shown in Table 4. It is noted from the table that the generalized *F*-test tends to reject the null hypothesis. Even in comparing the means A, B, C, D, E, F, and G, the generalized *F*-test suggests that we have strong evidence to reject the null hypothesis although the data are generated with the hypothesis being true. Nevertheless, *p*_SP_'s and *p*_PB_ are all larger than 0.05, which indicates that we cannot reject any null hypothesis. Thus, compared with the generalized *F*-test, the proposed tests in this paper provide more credible conclusions in detecting the significance of mean differences.

## 6. Concluding Remarks

This paper discusses a one-way ANOVA model with equicorrelation error structures. Hypotheses testing of the equality of the means and simultaneous confidence intervals for all pair-wise differences of the means are discussed. Numerical evidences indicate that the proposed tests perform much better than the generalized *F*-test provided by Lin and Lee [11], and the proposed simultaneous confidence intervals have good frequentist properties.

Although the generalized inference approach does not rely on any approximation, it often possesses frequentist properties only in the asymptotic sense (Hannig et al. [15], Xiong and Mu [19], and Xiong [20]). Krishnamoorthy et al. [14] and this paper show that the generalized *F*-test has no satisfactory performances in some cases. It may be a valuable issue to construct better tests for linear models with complicatedly dependent structures in the future.

## Figures and Tables

**(a):** 

*I* = 6, *ρ* = 0	*n*_*a*_			*n*_*b*_			*n*_*c*_			*n*_*d*_		
(*σ*_1_, *σ*_2_, *σ*_3_, *σ*_4_, *σ*_5_, *σ*_6_)	GF	SP	PB	GF	SP	PB	GF	SP	PB	GF	SP	PB
(1.0, 1.0, 1.0, 1.0, 1.0, 1.0)	0.078	0.037	0.041	0.067	0.043	0.049	0.055	0.042	0.050	0.062	0.047	0.058
(1.0, 1.0, 1.0, 1.5, 1.5, 1.5)	0.075	0.034	0.055	0.068	0.046	0.042	0.056	0.041	0.048	0.065	0.045	0.059
(1.0, 1.0, 1.5, 1.5, 2.0, 2.0)	0.068	0.034	0.052	0.066	0.042	0.038	0.063	0.046	0.045	0.063	0.043	0.048
(1.0, 1.5, 2.0, 2.5, 3.0, 3.5)	0.073	0.033	0.040	0.070	0.046	0.040	0.060	0.044	0.046	0.064	0.046	0.042

*n*_*a*_  =  (5,  5,  5,  10,  10,  10);  *n*_*b*_  =  (10,  10,  10,  10,  10,  10);  *n*_*c*_  =  (10,  10,  10,  15,  15,  15);  *n*_*d*_  =  (10,  10,  15,  15,  20,  20).

**(b):** 

*I* = 6, *ρ* = 0.5	*n*_*a*_			*n*_*b*_			*n*_*c*_			*n*_*d*_		
(*σ*_1_, *σ*_2_, *σ*_3_, *σ*_4_, *σ*_5_, *σ*_6_)	GF	SP	PB	GF	SP	PB	GF	SP	PB	GF	SP	PB
(1.0, 1.0, 1.0, 1.0, 1.0, 1.0)	0.078	0.037	0.043	0.067	0.043	0.049	0.055	0.042	0.051	0.062	0.047	0.057
(1.0, 1.0, 1.0, 1.5, 1.5, 1.5)	0.075	0.034	0.054	0.070	0.045	0.044	0.057	0.041	0.048	0.064	0.044	0.058
(1.0, 1.0, 1.5, 1.5, 2.0, 2.0)	0.069	0.033	0.052	0.068	0.044	0.040	0.064	0.047	0.047	0.063	0.041	0.047
(1.0, 1.5, 2.0, 2.5, 3.0, 3.5)	0.070	0.032	0.042	0.069	0.047	0.041	0.062	0.043	0.045	0.065	0.048	0.043

**(c):** 

*I* = 8, *ρ* = 0.1	*n*_1_			*n*_2_			*n*_3_		
(*σ*_1_, *σ*_2_, *σ*_3_, *σ*_4_, *σ*_5_, *σ*_6_, *σ*_7_, *σ*_8_)	GF	SP	PB	GF	SP	PB	GF	SP	PB
(1.0, 1.0, 1.0, 1.0, 1.0, 1.0, 1.0, 1.0)	0.085	0.042	0.053	0.080	0.045	0.056	0.071	0.047	0.056
(1.0, 1.0, 1.0, 1.0, 1.5, 1.5, 1.5, 1.5)	0.090	0.039	0.045	0.093	0.044	0.051	0.074	0.045	0.052
(1.0, 1.0, 1.5, 1.5, 2.0, 2.0, 2.5, 2.5)	0.087	0.037	0.042	0.084	0.039	0.047	0.072	0.041	0.046
(1.0, 1.5, 2.0, 2.5, 3.0, 3.5, 4.0, 4.5)	0.092	0.036	0.048	0.085	0.038	0.053	0.077	0.048	0.047

*n*_1_  =  (5,  5,  5,  5,  10,  10,  10,  10);  *n*_2_  =  (6,  9,  12,  15,  6,  9,  12,  15);  *n*_3_  =  (10,  12,  14,  16,  10,  12,  14,  16).

**(d):** 

*I* = 10, *ρ* = 0.3	*n*_4_			*n*_5_			*n*_6_		
(*σ*_1_, *σ*_2_, *σ*_3_, *σ*_4_, *σ*_5_, *σ*_6_, *σ*_7_, *σ*_8_, *σ*_9_, *σ*_10_)	GF	SP	PB	GF	SP	PB	GF	SP	PB
(1.0, 1.0, 1.0, 1.0, 1.0, 1.0, 1.0, 1.0, 1.0, 1.0)	0.094	0.045	0.057	0.096	0.045	0.053	0.076	0.044	0.051
(1.0, 1.0, 1.0, 1.0, 2.0, 2.0, 2.0, 2.0, 2.0, 2.0)	0.102	0.042	0.051	0.098	0.047	0.052	0.089	0.045	0.053
(1.0, 1.0, 1.0, 1.5, 1.5, 1.5, 2.0, 2.0, 3.0, 3.0)	0.094	0.040	0.043	0.083	0.041	0.047	0.077	0.045	0.047
(0.5, 1.0, 1.5, 2.0, 2.5, 3.0, 3.5, 4.0, 4.5, 5.0)	0.095	0.041	0.048	0.087	0.039	0.051	0.087	0.048	0.048

*n*_4_  =  (8,  8,  8,  8,  8,  10,  10,  10,  10,  10);  *n*_5_  =  (6,  8,  10,  12,  14,  6,  8,  10,  12,  14); *n*_6_  =  (10,  10,  10,  10,  10,  15,  15,  15,  15,  15).

**Table 2:** Coverage probabilities of the simultaneous confidence intervals.

*θ* = (1, 1, 2, 2)	*n* = (6, 8, 10, 12)		*n* = (10, 10, 10, 10)		*n* = (10, 15, 10, 15)	
*σ* = (*σ*_1_, *σ*_2_, *σ*_3_, *σ*_4_)	GP	PB	GP	PB	GP	PB
(1.0, 1.0, 1.0, 1.0)	0.958	0.943	0.957	0.948	0.956	0.954
(1.0, 1.0, 2.0, 2.0)	0.967	0.951	0.959	0.956	0.949	0.950
(1.0, 2.0, 1.5, 1.5)	0.972	0.943	0.955	0.948	0.959	0.946
(0.5, 1.0, 1.5, 2.0)	0.973	0.947	0.960	0.953	0.956	0.948

*θ* = (1, 2, 3, 4)						
*σ* = (*σ*_1_, *σ*_2_, *σ*_3_, *σ*_4_)						

(1.0, 1.0, 1.0, 1.0)	0.967	0.942	0.958	0.948	0.955	0.952
(1.0, 1.0, 2.0, 2.0)	0.959	0.945	0.962	0.949	0.946	0.947
(1.0, 2.0, 1.5, 1.5)	0.973	0.952	0.966	0.952	0.953	0.953
(0.5, 1.0, 1.5, 2.0)	0.964	0.945	0.971	0.950	0.956	0.948

**Table 3:** Summary statistics in Section 5.

Treatments	*n*_*i*_	*σ*_*i*_	*ϕ*_*i*_	${\hat{\mu }}_{i}$	*λ*_*i*_^2^
A	5	1.0	5.60	1.806	12.835
B	5	1.5	7.60	2.053	40.928
C	5	2.0	10.40	0.179	10.731
D	5	2.5	14.00	1.492	32.809
E	10	3.0	18.40	0.369	111.134
F	10	3.5	23.60	1.995	430.336
G	10	4.0	29.60	−1.163	290.230
H	10	4.5	36.40	0.743	653.191

**Table 4:** *P* values in Section 5.

Treatments compared	*P*_GF_	*P*_SP_	*P*_PB_
A, B, C, D, E, F, and G	0.021	0.091	0.075
A, B, C, D, E, F, and H	0.111	0.234	0.186
A, B, C, D, E, H, and G	0.032	0.109	0.134
A, B, C, D, F, G, and H	0.036	0.105	0.115
A, B, C, E, F, G, and H	0.039	0.101	0.089
A, B, D, E, F, G, and H	0.159	0.151	0.152
A, C, D, E, F, G, and H	0.061	0.124	0.073
B, C, D, E, F, G, and H	0.106	0.212	0.182

## References

[1] Tsui KW, Weerahandi S (1989). Generalized *p*-values in significance testing of hypotheses in the presence of nuisance parameters. *Journal of the American Statistical Association*.

[2] Weerahandi S (1993). Generalized confidence intervals. *Journal of the American Statistical Association*.

[3] Weerahandi S (2004). *Generalized Inference in Repeated Measures*.

[4] Weerahandi S (1995). ANOVA under unequal error variances. *Biometrics*.

[5] Ananda MMA, Weerahandi S (1997). Two-way ANOVA with unequal cell frequencies and unequal variances. *Statistica Sinica*.

[6] Gamage J, Weerahandi S (1998). Size performance of some tests in one-way ANOVA. *Communications in Statistics B: Simulation and Computation*.

[7] Bao P, Ananda MMA (2001). Performance of two-way anova procedures when cell frequencies and variances are unequal. *Communications in Statistics B: Simulation and Computation*.

[8] Mu W, Xiong S, Xu X (2008). Generalized confidence regions of fixed effects in the two-way ANOVA. *Journal of Systems Science and Complexity*.

[9] Chi EM, Weerahandi S (1998). Comparing treatments under growth curve models: exact tests using generalized *p*-values. *Journal of Statistical Planning and Inference*.

[10] Weerahandi S, Berger VW (1999). Exact inference for growth curves with intraclass correlation structure. *Biometrics*.

[11] Lin S, Lee J (2003). Exact tests in simple growth curve models and one-way ANOVA with equicorrelation error structure. *Journal of Multivariate Analysis*.

[12] Ho Y-Y, Weerahandi S (2007). Analysis of repeated measures under unequal variances. *Journal of Multivariate Analysis*.

[13] Mu W, Xu X (2012). Inference for repeated measures models under heteroscedasticity. *Journal of Systems Science and Complexity*.

[14] Krishnamoorthy K, Lu F, Mathew T (2007). A parametric bootstrap approach for ANOVA with unequal variances: fixed and random models. *Computational Statistics & Data Analysis*.

[15] Hannig J, Iyer H, Patterson P (2006). Fiducial generalized confidence intervals. *Journal of the American Statistical Association*.

[16] Xiong S, Mu W, Xu X (2008). Generalized inference for a class of linear models under heteroscedasticity. *Communications in Statistics: Theory and Methods*.

[17] Efron B (1979). Bootstrap methods: another look at the jackknife. *The Annals of Statistics*.

[18] Xiong S, Mu W (2009). Simultaneous confidence intervals for one-way layout based on generalized pivotal quantities. *Journal of Statistical Computation and Simulation*.

[19] Xiong S, Mu W (2009). On construction of asymptotically correct confidence intervals. *Journal of Statistical Planning and Inference*.

[20] Xiong S (2011). An asymptotics look at the generalized inference. *Journal of Multivariate Analysis*.

